# “It shreds me from within”: thematic analysis of the impact of racism on veterinary professionals and students in the United Kingdom

**DOI:** 10.1186/s40359-025-02481-x

**Published:** 2025-05-31

**Authors:** Naomi King, Jacqueline M Cardwell, Elisa G. Lewis, Nimisha Rajesh Patel-Cook, Christine Thuranira-McKeever, Victoria J. Crossley, Navaratnam Partheeban

**Affiliations:** 1 Royal Veterinary College, Hawkshead Lane, North Mymms, Hatfield, Hertfordshire AL9 7TA UK; 2 London South Bank University, 103 Borough Road, London, SE1 0AA UK; 3 British Veterinary Ethnicity and Diversity Society, 31/6 Marchmont Crescent, Edinburgh, EH9 1HQ UK; 4 Present Address: Centre for Psychological Research, Oxford Brookes University, Oxford, UK; 5 Present Address: Imperial College, Exhibition Road, London, SW7 2AZ UK

**Keywords:** Racism, Ethnicity, Black, Asian, and minority ethnic (BAME), Veterinary profession, Identity Process Theory, Identity conflict

## Abstract

**Background:**

Calls to tackle racism, especially in the workplace, have risen recently around the world and much research has examined the experiences of Black, Asian, and minority ethnic (BAME) people in professions with relatively high ethnic diversity. Far less has investigated these experiences in low ethnic diversity professions, however, where BAME people may face a different and more damaging range of challenges in a context where racism is often overlooked. This qualitative study advances existing understanding of this topic by examining the psychological impact of racism on BAME people in one of the United Kingdom’s (UK’s) least ethnically diverse professional groups: the veterinary sector.

**Methods:**

All BAME people working or studying in any part of the UK veterinary sector were invited to complete an online questionnaire disseminated via veterinary schools, organisations, social media, and other veterinary media. The questionnaire included two open questions that asked participants to describe: (i) any incidents regarded as racist that they had witnessed or experienced in the context of the veterinary sector, and (ii) how they felt, how they dealt with the incidents, and any impact the incidents had on their wellbeing, mental health, or job satisfaction. The data were analysed using reflexive thematic analysis, with a combination of inductive (data-driven) and deductive (theory-driven) approaches, underpinned by a critical realist ontology and a contextualist epistemology.

**Results:**

Analysis produced three main themes: *Alienation and insecurity*, *Homogenisation and inferiorisation*, and *Disappointment and futility*, with an overarching theme of *Identity conflict*. Drawing on Identity Process Theory (IPT), the findings showed how experiencing racism in the workplace undermined some or all of the participants’ six ‘identity principles’ that IPT proposes are necessary to construct and maintain a positive sense of identity: *continuity, distinctiveness, self-efficacy, self-esteem, belonging,* and *meaning*. In addition, racism altered participants’ sense of identity in undesirable ways on both interpersonal and individual levels.

**Conclusions:**

These findings highlight an urgent need to acknowledge and address racism in low ethnic diversity professions, and offer new insight into the psychological consequences of systemic, societal racism on a broader scale.

**Supplementary Information:**

The online version contains supplementary material available at 10.1186/s40359-025-02481-x.

## Background

Calls to tackle racism have risen recently worldwide, especially in the United Kingdom (UK) and the United States (US), exemplified by the resurgence of the Black Lives Matter movement in 2020 [1] and international reports from influential bodies such as the United Nations [2] and Amnesty International [3]. Professions and organisations have come under increased scrutiny for how they handle racism, and many have committed to anti-racist policies and active anti-racist work [4, 5]. Efforts to centralise issues of race and racism have led to counter-responses that deny their extensiveness and relevance, however, making them relatively invisible in a wide range of contexts [5]. In spite of the increased calls for action, a significant proportion of UK society seems unaware of or unconcerned with racism and its effects, and many professions and organisations appear to lack the necessary understanding, will, and power to address it in meaningful ways [4].

While much research has examined the experiences of Black, Asian, and Minority Ethnic (BAME)^1^ people in professions with relatively high ethnic diversity, such as medicine, other healthcare professions, and higher education (e.g., [6, 7]), far less has investigated these experiences in low diversity professions. This is problematic, as the findings and suggestions for interventions from one group are not necessarily transferable to the other. It is more likely, for example, that people working in low diversity professions will be seen as different by colleagues and clients, judged in terms of their ethnicity, subjected to conscious or unconscious discrimination, find it difficult or impossible to talk about racism, and lack colleagues who fully understand racism and its consequences or managers who consistently support them and take decisive action. It is also more likely that white people in such professions will believe that institutional racism is a problem of the past and irrelevant to their workplaces, a view that the UK government’s ‘Sewell report’ [8] has been criticised for taking [9].

A diversity ranking of occupations in England and Wales [10] indicates that the veterinary profession incorporates two of the UK’s least ethnically diverse professional groups: veterinary surgeons and veterinary nurses. BAME people were estimated to form around 3.5% of veterinary surgeons and 1.9% of veterinary nurses registered with the UK’s Royal College of Veterinary Surgeons (RCVS) in 2019 [11, 12], compared to 44.4% of NHS medical staff in England [13], and an estimated 14.4% of the UK’s 2019 general population [14]. In this article, the term ‘veterinary profession’ is used to encompass veterinary surgeons, veterinary nurses, and students of both.

Although racism experienced by veterinary professionals has recently been highlighted in the UK [15], the US [16], and Canada [17], with two British Veterinary Association (BVA) surveys [15] finding that over a quarter of UK BAME veterinary staff and students had personally experienced racism over the previous year, an extensive literature search preceding our study found just one relevant UK-based peer-reviewed publication [18]. Chung and Armitage-Chan reported analysis of focus group data from 25 UK BAME veterinary students, all of whom spoke about racism encountered at university and/or during external placements [18]. Many experienced a sense of alienation, a lack of confidence in reporting the incidents, and low awareness from white peers of how different BAME students’ university experiences were.

Anecdotal accounts of racism experienced by UK veterinary professionals are brief and lack formal analysis (e.g., [19–22]). In the most detailed of these, Limb presented three case studies of BAME veterinarians describing a wide range of racism received from clients, students, colleagues, and managers [20]. They highlighted a lack of support from white colleagues, and the very real risk of backlash and repercussions that prevented BAME people from speaking out. Psychological consequences included frustration, unease, damaged confidence, a strong sense of isolation, depression, and mental and emotional exhaustion.

These findings must be taken in the context of a profession widely acknowledged to be challenging and demanding, with veterinarians around the world showing elevated levels of stress, mental health problems, burnout, and suicide [23–25]. In the UK, the proportional mortality ratio for suicide among veterinarians is estimated to be three to four times the national average [24], and approximately twice that of medical and dental practitioners [26]. Proposed reasons for these issues include long and antisocial working hours, uncomfortable working conditions, poor work-life balance, distressed or difficult clients, moral and ethical dilemmas, and compassion fatigue [23, 25]. BAME people working in the profession are therefore confronted with a multitude of additional stressors alongside racism, which may diminish the high levels of motivation and determination needed to thrive and succeed in their work.

Many UK veterinary professionals also conduct at least some of their work in rural locations [11, 12]. This is typical of low diversity occupations but not high diversity occupations; non-white minorities in England and Wales are estimated to form only 0.2% of gardeners and landscape gardeners, 0.3% of farm workers, 0.6% of environment professionals, and 0.8% of farmers [10]. Population estimates for 2020 showed that BAME people accounted for approximately 3.2% of England’s rural population, decreasing to 1.5% in sparsely populated areas, compared to 18.3% in urban areas [27]. Studies have found that white people in rural England and Scotland tend to have little contact with BAME people and consciously or unconsciously hold a wide range of prejudicial stereotypes, perceiving them as a threat to cultural homogeneity; unsurprisingly, this often makes BAME people feel incongruous and out of place [28–31]. Chakraborti [28] noted that although some BAME households in rural England reported explicit racial abuse and even physical violence, many felt it was the “cumulative ‘drip-drip’ effect” of more subtle forms of racism that had the most significant and corrosive effect (p. 506).

Together, these findings highlight an urgent need for further investigation into BAME people’s experiences of racism in low ethnic diversity professions, and its impact on their wellbeing and mental health. This is essential for developing tailored interventions and monitoring their effectiveness. The veterinary profession can be considered an optimal exemplar in this instance, as it is not only a profession with low ethnic diversity but one in which other significant barriers for BAME people intersect. The primary aim of this study is to advance understanding of the psychological effects of racism on BAME people in such professions, including the subtle and ambiguous forms of racism that often go unnoticed and unchallenged.

## Methods

### Survey

We created an online survey comprising a participant information sheet and statement of consent followed by three sections of questions: eight demographic questions, six multiple choice questions, and four open-text questions. The full survey, developed specifically for this study, is available in the Supplementary Materials. This paper presents an analysis of responses to the initial two open-text questions. The first was: *Please describe in detail any racist incidents you have witnessed or experienced in the context of the veterinary sector. Relevant details might include, for example, the types or roles of the people involved, when (how long ago) and where the incident(s) occurred, whether you reported the incident(s) or sought support, and the outcome of any report(s) made.* The second was: *Please describe how you felt, how you dealt with these incident(s), and any impact this has had on your wellbeing, mental health, or job satisfaction.* The introduction to the open-text section emphasised that the researchers were interested in anything that respondents regarded as racism, including everyday racism, microaggressions, or other common behaviours, as well as more extreme incidents. The information sheet signposted appropriate sources of support, in case the relating of racist incidents caused distress, and advised participants that they were free to withdraw from the study for up to four weeks after submitting their responses. As all data were anonymous, we required participants to enter a unique personal code so that their data could be identified should they wish to withdraw it. The study was approved by the Social Science Research Ethical Review Board of the Royal Veterinary College (URN SR2020-0224).

### Procedure

Prior to release, we piloted an initial version of the survey comprising the demographic and open-text questions with 12 volunteers recruited via the British Veterinary Ethnicity and Diversity Society network, six of whom answered the first two open-text questions. Subsequently, we added the statement ‘*Please write as much as you are comfortable to – including detail helps with our qualitative analysis. There is no word limit’*. We then piloted a second version of the survey comprising the demographic, multiple-choice, and open-text questions with a further 14 participants, seven of whom answered the first two open-text questions. Following this, we made these two questions mandatory. The final survey was open from 19^th^ January to 31^st^ March 2021. We disseminated a link to the survey via UK veterinary schools, veterinary organisations and groups, social media, and other veterinary media. All BAME people working or studying in any part of the UK veterinary sector were invited to participate.

### Participants

We selected a subsample of 69 participants, including nine pilot participants, from a total of 258 who had proceded beyond the demographics section, 175 of whom had answered the first two open-text questions. Sixty (87%) participants were female, reflecting the predominantly female source populations [11, 12]; see Table 1 for full demographic details. Inclusion criteria were that participants had (a) written at least 170 words for the two open-text questions combined, and (b) self-identified as BAME, rather than any form of solely white ethnicity.^2^ Ethnic group categories were based on those used in the 2011 Census of England and Wales [32]. The minimum word count was determined by author NK successively reviewing all responses of ≤ 50 words, ≤ 100 words, ≤ 125 words, ≤ 150 words, ≤ 170 words, and > 170 words to judge the coherence, consistency, detail, and depth of the information conveyed. Through discussion, all authors agreed that only responses of at least 170 words met these four conditions and contained sufficiently rich data for a reflexive thematic analysis. Responses for the two questions combined ranged from 170 to 1,564 words, and all recounted personal experiences of racism in the UK veterinary sector.

**Table 1:**
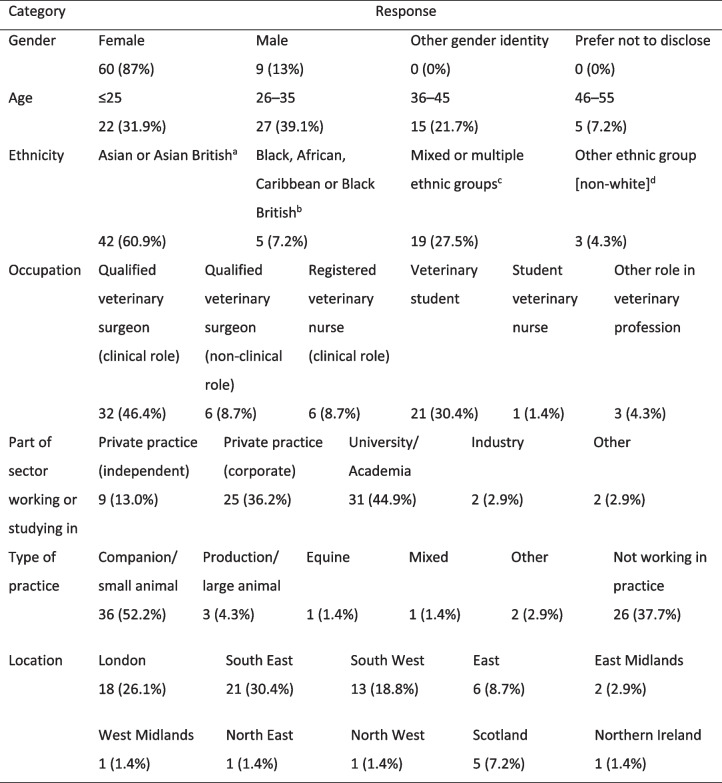
Demographic characteristics of 69 questionnaire participants who provided free-text responses to questions about their experiences of racism while working or studying in the UK veterinary profession

^a^ Indian; Pakistani; Bangladeshi; Chinese; Any other Asian or Asian British ethnicity

^b^ African; Caribbean; Any other Black or Black British ethnicity

^c^ White and Black Caribbean; White and Black African; White and Asian; Any other mixed or multiple ethnicity

^d^ Arab or British Arab; Any other ethnic group

### Data analysis

We used reflexive thematic analysis (RTA) [33–35], an approach that acknowledges the active role of researchers in analysis and the influence of their subjective perspectives and interpretations. The analysis was primarily conducted by author NK, but all documents were shared among authors and stored securely as part of an audit trail [36]. Regular online meetings and the diversity of the team allowed multiple perspectives to be incorporated into data interpretation. Three members of the team (CTM, NRPC, and NP) are BAME members of the UK veterinary profession, contributing interpretations based on lived experience. CTM and NP also brought experience from leadership roles in the field of equity, diversity, and inclusion. NK, JMC, EGL, and VJC are white; NK, JMC and EGL are experienced in qualitative data analysis; and JMC and VJC are members of the UK veterinary profession. All members of the team therefore brought different personal perspectives to the analysis, but with the combined intention of privileging participants’ accounts. We assumed a relativist ontology (i.e., reality is a subjective experience) and constructivist epistemology (i.e., people construct their own meaning, based on their experiences).

NK read each transcript and entered raw data into the left-hand column of a table. She then created an extensive set of descriptive and interpretive notes in the middle column, highlighting points of interest based on an iterative process of repeated reading and reflection, and developed corresponding codes in the right-hand column. Codes ranged from brief summaries of surface meanings (semantic) to deeper interpretations of underlying, subliminal content (latent). For example, a recurrent semantic code was ‘participant referred to by ethnicity, not by name’; latent interpretations of this included ‘participant’s name and identity not valued or respected’, ‘BAME vets anonymised and homogenised’, and ‘BAME vets categorised as outsiders who do not belong in the UK’. The analysis at this stage was inductive (data-driven), meaning that codes were generated based on the data, without deliberate imposition of any pre-existing theories (i.e., accepted explanatory frameworks that can be used to guide data interpretation).

After an iterative process of code refinement and collation of the entire set of codes, tentative themes were developed collaboratively, through team discussions, by clustering codes that were related by a shared meaning rather than simply by topic. This was informed both by NK’s original reflections and by interpretive input from other team members. As our theme structure progressed, NK suggested that, based on a data-led exploration of the literature, application of Identity Process Theory (IPT) (e.g., [37–42]) would offer significant insight into participants’ experiences. For this reason, the analysis shifted from an inductive approach to a combination of inductive and deductive (theory-driven) approaches [33, 34], as we subsequently used IPT to guide our ongoing theme construction and refinement.

IPT proposes that six ‘identity principles’ are necessary for construction and maintenance of a positive sense of identity. These are *continuity* (feeling that one’s identity is consistent across time and contexts); *distinctiveness* (feeling unique and distinct from others); *self-efficacy* (feeling confident and capable); *self-esteem* (feelings of self-worth and self-respect); *belonging* (feeling included and accepted by others); and *meaning* (feeling one’s life has significance and purpose) [37, 38, 41, 42]. Deductive application of IPT to our data involved aligning our inductively developed code clusters with these principles. For example, clusters of codes relating to participants’ feelings of alienation aligned with the IPT principles of *belonging* and *continuity*. The final theme structure is outlined in detail in the results section.

Themes were systematically reviewed and revised to ensure that each had ‘internal homogeneity’ and ‘external heterogeneity’ [33, 43], and cross-checked against the original data to ensure that they were firmly grounded in participants’ reported experiences. We assigned randomly-ordered numerical identifiers (P01–P69) to participants, and amended minor typographical and grammatical errors for readability in the quotes presented below. We have not presented demographic details for the quoted participants, to avoid compromising anonymity in the context of a small, predominantly white profession.

## Results

**Fig. 1:**
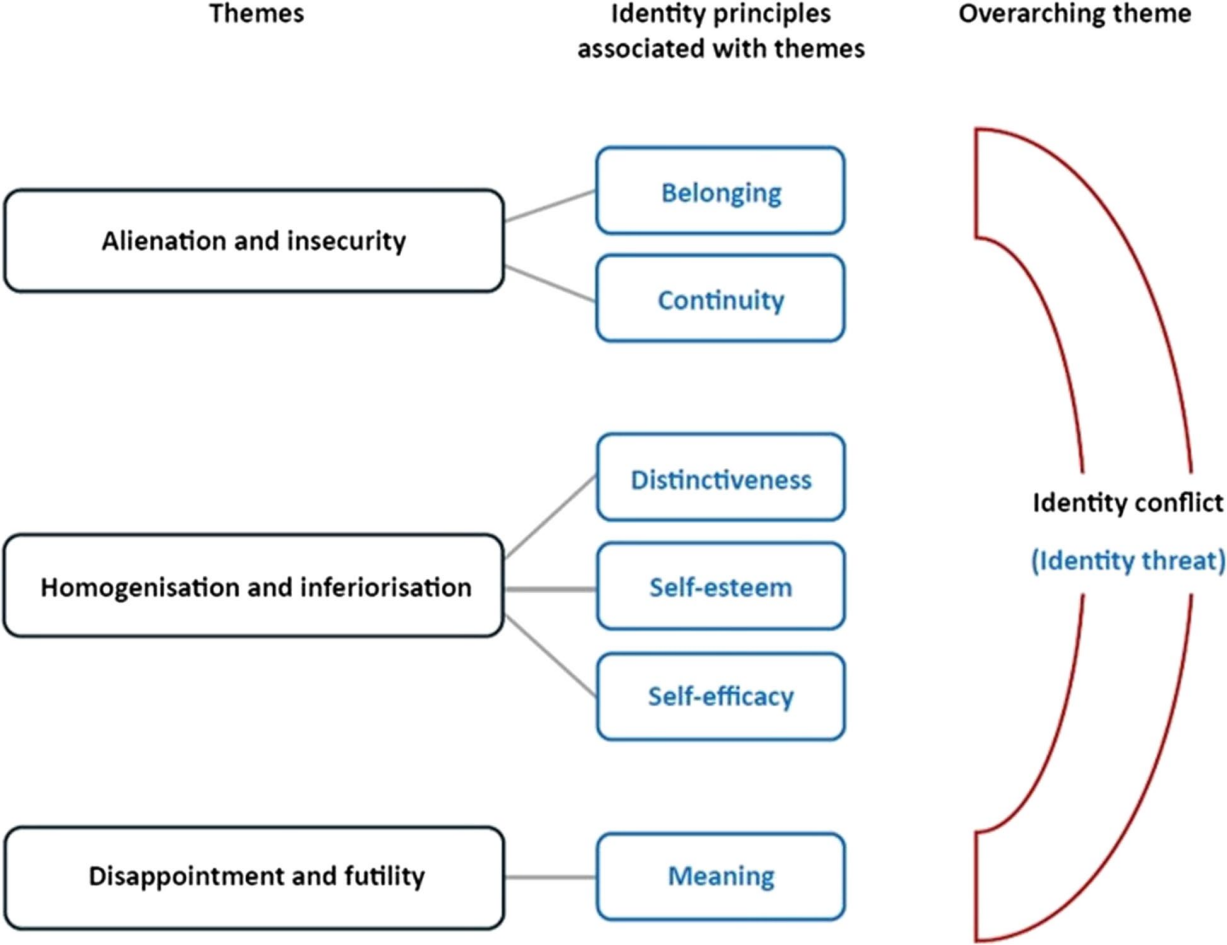
Themes (aligned with identity principles from Identity Process Theory) constructed through reflexive thematic analysis of 69 open text responses from veterinary professionals and students to two survey questions about the nature and impact of racist incidents experienced in the UK veterinary sector

This section comprises three themes: (1) *Alienation and insecurity*, (2) *Homogenisation and inferiorisation,* and (3) *Disappointment and futility*. These themes reflect the initial inductive code patterns, but each also aligns with one or more of the six ‘identity principles’ of IPT (Fig. 1). A fourth, overarching theme of *Identity conflict*, defined by IPT as a form of identity threat, runs through each of these three themes and is addressed in the general discussion.

### Alienation and insecurity

Many participants experienced a sense of exclusion and alienation in the veterinary sector, frustrating their need for *belonging*. They described various white people, including clients, colleagues, managers, student peers, placement hosts, and lecturers, speaking or acting in ways that indicated how they perceived BAME people to be different, strange, and foreign, due to their ethnicities. Participants felt ‘isolated’ (P04, P45, P46, P50, P62), ‘alone’ (P27, P45, P54), like an ‘outsider’ (P26, P61), ‘out of place’ (P37, P50, P65), and ‘not welcome’ (P01) in the profession. Some recounted incidents of blatant rejection:

In practicals in the vet school, I’ve noticed more and more times that students would rather work on other stations and not want to work on stations me and my friends (who are also from ethnic minority backgrounds) are working on, even when the other stations are full. This has happened on multiple occasions. (P16)

More frequently, participants described being ‘othered’ in less overt ways. White people often asked questions that suggested that BAME people could not be British due to their skin colour, an assumption that usually conflicted with the participant’s sense of self:

People assume that you aren't British and are different, whereas I would say since I was born and grew up in Britain that I identify as British. […] People ask me […] if I can speak another language, or like, do I eat my ethnic food all the time? (P65)

While some participants thought that such questions were based on genuine ignorance, others regarded the interrogators as being deliberately patronising and estranging. White people sometimes refused to believe that the participants were the same as ‘normal’ British people:

[On] work experience, […] the vet I was shadowing at the time would ask me questions like, “Are you integrated into society yet?”, and “What do you eat?”. I am a British citizen who was born in the UK, and so were my parents, so I found this particularly confusing. I would reply, “I eat the same kind of foods as everyone else”, [but] the vet would continue to ask questions like, “No, but what do you have for dinner?”. […] [It] made me feel quite upset. (P41)

A number of participants related being asked where they were from:

An older couple […] once probed, “Where are you from?”, I said [name of UK city], they asked again, I said I grew up in [name of UK county], and they asked one more time and I said I have Caribbean heritage. I find this is a common form of racism amongst older people and often they’re not trying to be hurtful, but they’re curious and don't understand how it would make someone feel uncomfortable. (P30)

This ‘where are you from?’ question is a common form of ‘everyday’ racism, highlighted by Sue, Capodilupo, et al. as a ‘microinvalidation’ that makes BAME people feel ‘alien in [their] own land’ ([44], pp. 281–282). Unlike more explicit forms of racism, microinvalidations communicate implicit racist attitudes and beliefs that ‘exclude, negate or nullify the psychological thoughts, feelings or experiential reality’ of BAME individuals ([45], p. 73). Although these participants believed that most people who asked them where they were from did not intend to be offensive, the assumption that BAME people were ‘foreigners’ caused feelings of discomfort and unease.

Many participants’ experiences in the veterinary sector had made them more aware of the gap between their own personal and cultural values and the ‘racially biased worldview’ held by many white people ([46], p. 331). Ignorance about what constituted racism, or unwillingness to learn more about it, meant that white people could not understand what BAME people experienced, or empathise with their feelings. Such attitudes were frequently expressed by clients, reflecting a deep undercurrent of racism in UK society more generally. However, they were also sometimes voiced by professional colleagues or student peers:

[A casual racist remark from a junior colleague] […] made me realise how much subconscious and internalised racism there was, even in people who weren’t ever overtly racist. This is something that still makes me very sad. (P54)

Many participants also experienced a strong sense of estrangement when white people failed to support them or stand up for them in the face of racism. This had a long-lasting effect on their perceptions of their workplaces, universities, and communities:

What hurt the most [after an overt racist incident from a stranger] was that not a single person in the practice said anything or defended me, including my own team. I was so shaken and no one showed any support. […] I felt disgusted, hurt, and really disappointed in my colleagues and my community. (P09)

For some participants, their overall lack of a sense of *belonging* was linked to a loss of their sense of *continuity*. For a few veterinary students, entering the profession led to a sudden and disturbing revelation:

By all these instances [of experiencing subtle racism as a student], I was made to feel ‘different’ and became blindingly aware of how ‘different’ I am perceived as. I find this difficult as I was born in the UK and see myself as British. (P35)

For other participants, it was a slower and more long-term process whereby cumulative effects of various small racist incidents in the profession gradually eroded their sense of identity. They could no longer be who they thought themselves to be:

Most of the time during the incident I barely even notice it has happened or that someone has said something racist or inappropriate. But over time it has made me very insecure in my identity and made me feel that I don't belong. […] I don't see the profession ever feeling like somewhere that I belong – I will always be an outsider. (P26)

This feeling often extended beyond the veterinary profession and significantly influenced other aspects of participants’ lives:

I had never thought that I would be assumed non-British by a stranger until [two vets separately indicated they thought otherwise]. […] It just felt like my identity, which is British, was overlooked, ignored, and removed. For the first time, it made me consider how others saw me and I felt very strange about it. It occupied my thoughts a lot. (P58)

Some participants felt obliged to modify or change themselves in order to ‘fit in’ with white people’s expectations:

The microaggressions make you feel […] that sometimes you have to ‘fit in’ and change yourself a bit to fit the mould of what people expect and want, but you sometimes feel a bit disheartened because at the end of the day you can't change the way you look or really how you feel. (P65)

This led to internal conflict and a pervasive feeling of insecurity, as the participants realised that their sense of self was being pulled in opposing directions.

### Homogenisation and inferiorisation

Although many participants thought they were generally seen as different from white people (difference *between* groups), they also believed they were seen as very similar to other BAME people (lack of difference *within* BAME groups). This thwarted their need for individual *distinctiveness*, a positive sense of being special and unique that is necessary for wellbeing [47, 48]. Instead of using their names, clients or colleagues regularly referred to participants or their BAME colleagues by what they (often incorrectly) assumed to be their ethnicities:

I've had lay colleagues refer to me as ‘the Chinese vet’ in front of clients and other colleagues rather than address me by my name – and it's weird because I grew up in Australia. (P08)

Such comments objectified and deindividualised the BAME person, but white people did not seem to see their behaviour as problematic. This was highlighted by one participant’s experience on a placement:

Clients often […] asked to book in with the Singapore vet, but commonly referred to him as ‘the Chinese vet’ on the phone instead of his name, or if they forgot his name. I believe the nurses corrected these clients, but sometimes the clients would ‘jokingly’ comment that it's ‘the same thing’. (P58)

Some participants also related how clients, colleagues, and student peers did not make the effort to try to pronounce their names properly, and sometimes acted as if such ‘foreign’ names were appropriate targets for humour or ridicule:

Many of my colleagues don't try to pronounce my last name just because it's a foreign name. I have been working for a year and they always just laugh off that they can't pronounce it. I find it a bit rude. (P60)

Others were given nicknames that they found aversive. Their real names and identities were ‘whitewashed’ for the convenience of colleagues and clients, erasing their true identities and forcing them to conform to white cultural norms:

At [one veterinary practice], the staff gave me a nickname rather than call me by my first name as it was difficult for them to pronounce. […] It was upsetting to be given a nickname that I hadn't made for myself, as I am proud of my name and my heritage. It felt like I was being whitewashed for convenience. (P37)

Several participants recounted how white people often mixed them up with other BAME colleagues or students:

[My Black colleague and I] have totally different names, we look different, we work in different locations AND have two different job roles. For some reason, when I first started, a couple of people would mix us up. […] The mix-up even came to a point where my managing director messaged my other Black colleague instead of me. (P51)

This inability to distinguish between BAME people was often brushed off by white people as a simple error, but seemed to reflect a common racist belief that people of a given race are all alike [45], as articulated clearly to one veterinary student on a farm placement:

The farmer’s daughter was discussing a particular Black athlete with the other vet student, [and] the farmer commented, “All these Blackies look the same to me”. The farmer’s daughter and the other vet student burst out laughing and the daughter then said jokingly, “That’s a bit racist, dad”. I said, “Aren’t you stereotyping a whole race and discrediting a person’s individuality?” […] and the farmer then shut me down by saying, “Oh pipe down you, we weren’t talking about you!”. (P54)

Being seen ‘only through the lens of race’ (P46) did not merely diminish the participants’ sense of individuality and uniqueness. Many described how white people frequently applied well-established negative stereotypes to BAME people, which had a harmful effect on their need for *self-esteem* and *self-efficacy.* These stereotypes were sometimes communicated by small, casual comments and insinuations:

One of our nurses said that her dad used to joke that her mum might run off with a Black man. This made me feel uncomfortable, as the insinuation was that […] being Black was a bad thing. […] I thought it odd that she should repeat it in front of me. (P28)

On the other hand, however, such stereotypes were sometimes revealed through explicit racial abuse:

[A farmhand on a student placement] would make a lot of [racist] remarks about me behind my back and would be incredibly rude and stand-offish when we had to work together. […] It was really upsetting to be judged and ostracised based [on] ignorant assumptions. […] [He] wouldn’t allow me to participate in certain tasks, [and] would call me lazy and useless. […] It was really dehumanising. (P63)

These incidents damaged the participants’ self-confidence, self-worth, and self-respect. They reported feeling ‘inferior’ (P40, P47, P51, P67), ‘inadequate’ (P29, P65), ‘helpless’ (P04, P06, P51, P52), and humiliated:

I feel like all these incidents do impact my wellbeing and mental health, as often they leave me feeling either humiliated or more anxious about my circumstances. Sometimes it even discourages me from speaking to people, which impacts my self-esteem and courage to speak up for myself. (P69)

Some female participants thought the intersection between ethnicity and gender rendered them doubly inferior in the eyes of their clients:

It’s hugely upsetting, undermines my hard work and dedication in this field, that I have to fight extra hard as an Asian female veterinarian to get the same level of understanding, respect, and appreciation as my other colleagues. (P44)

Many participants described both clients and colleagues communicating stereotypical perceptions of BAME people having lower intelligence, competence, and skills [49, 50], with clients sometimes refusing to receive their service on these grounds, and even likening it to ‘taking your car to a bad mechanic’ (P03). This often left participants feeling that they needed to prove their worth by working harder than their white colleagues:

Clients […] stated behind my back that I must be a charity case for the university as my people don't belong in science fields or in leadership roles. […] I feel like I have to constantly prove my worth. (P18)

As well as experiencing various levels of weariness and exhaustion, such participants found themselves trapped in a vicious circle; trying to invalidate and disprove a low-ability stereotype creates an extra cognitive and emotional burden of effort and stress, which in turn can ultimately decrease performance [49, 50].

### Disappointment and futility

Underlying many of the participants’ experiences was an overall loss of *meaning* in their lives. They felt ‘disappointed’ (P09, P38, P48, P54), ‘disheartened’ (P41, P65), and ‘dispirited’ (P41), as racism resulted in lower levels of fulfilment in their work or studies:

Job satisfaction worsens when I learn that my colleagues (nurses or vets) are unaware [of] the impact of their words and do not understand what constitutes racism, e.g., use of the word ‘golliwog’, even when not used to describe a specific person. […] The judgement placed on someone due to their ethnicity creates an unpleasant atmosphere which impacts job satisfaction. (P03)

A few veterinarians had considered leaving the profession, as the ‘massive impact’ (P47) of racist incidents meant that they no longer felt their careers to be rewarding or meaningful. Some students wondered whether they should even enter the profession:

After [racist incidents during] the lambing placement, I did question whether I still wanted to be a vet or if I wanted to go into another profession where these situations would be less likely to happen. […] It has made me question my place in the veterinary profession and what challenges I may potentially face when I qualify. (P16)

The accumulation of these racist incidents, sometimes ‘almost imperceptible’ (P25) and ‘so subtle, you question whether they really happened’ (P54), led many participants to experience ongoing feelings of frustration and futility. On a deeper level, racism experienced in the veterinary sector permeated many participants’ lives beyond the workplace, sometimes causing them to question their own personal value and significance:

Each incident has an impact; sometimes it feels like a little sting, other times it feels like a ton of bricks has landed on you. There is a cumulative effect that changes your view of yourself and where you see yourself in society, how worthy you are of a place in this world. (P17)

Several participants explained how racism had created or exacerbated mental health problems, including chronic stress, depression, burnout, imposter syndrome, and suicide ideation. One described their mental suffering in particularly vivid terms:

The ongoing battle to prove myself to others has put a constant pressure on me. It basically is a part of my personality. It drains a lot of energy and at times it overwhelms me. The worst part is that there is no end to it and I suffer from depression. I always wonder what if I was free from this constant pressure and free from the desire to prove this or that, would I be free from depression?! This is what racism has done to me. Made me a strong fighter but it shreds me from within, and whilst I have achieved high and overcome obstacles and barriers, it has weakened my mind, drained my energy, and I live in a constant battle with racism and depression. (P36)

This ‘ongoing battle’ reflects many other participants’ accounts of continually trying to prove themselves in the veterinary sector, but also reveals in striking depth the psychological damage inflicted by racism over time. Life for this participant had become an endless Sisyphean task that was both extremely effortful but ultimately pointless, as there seemed to be no way in which the larger battle against racism could ever be won.

## Discussion

This study sought to gain in-depth understanding of the psychological effects of racism on BAME people working or studying in the UK veterinary sector, as an exemplar of low ethnic diversity professions, furthering the minimal amount of existing literature on this topic. On a descriptive level, the findings support and expand on those of the BVA [15], Chung and Armitage-Chan [18], and Limb [20], showing that racism is a widespread problem encountered by BAME veterinarians, veterinary nurses, veterinary students, and other veterinary staff members; that the perpetrators include not only clients but colleagues, managers, academics, student peers, and placement hosts; and that racism in all forms, whether explicit or insidious, intentional or unintentional, has a negative impact on BAME people’s wellbeing and mental health within and beyond their workplaces. They also reflect the findings of other studies examining BAME people’s experiences in under-researched, low ethnic diversity UK occupations where racism is frequently overlooked, including policing [51, 52], academic and research librarianship [53], sports coaching [54], and public relations [55].

Subtle, ‘everyday’ racism was described most frequently by participants, with a smaller number reporting overtly offensive incidents. ‘Everyday’ racism is considered particularly problematic due to its ambiguous nature and the fact that it is so common and deeply embedded in predominantly white societies [44–46, 56]. It is typically overlooked, denied, or trivialised by white people, and as a consequence, BAME people often worry about being seen as oversensitive, petty, or paranoid [44, 45]. When they are directly accused of being so, this creates a considerable personal dilemma and may lead them to question their own interpretations of incidents, as many can seem unintended and insignificant at the time [44, 45]. This renders the cumulative effect doubly detrimental, and it contributed to insecurity and self-doubt in our participants.

On a more interpretative level, the theoretical framework of Identity Process Theory offered deeper insight into participants’ psychological experiences. IPT proposes that individuals continually seek to construct and maintain their identities through two ongoing, universal processes: *assimilation–accommodation* and *evaluation*. When new information (e.g., new knowledge, attitudes, beliefs, or behaviours) cannot be *assimilated* into an individual’s existing identity structure, their existing identity cannot be modified to *accommodate* the new information, and/or the new information is *evaluated* in a negative way, the person experiences identity threat and reduced wellbeing. These two processes are guided by six ‘identity principles’ that people strive to satisfy: *continuity*, *distinctiveness, self-efficacy*, *self-esteem* [37, 38], *belonging, and meaning* [41, 42]. Racism experienced while working or studying in the veterinary sector appeared to undermine some or all of these identity principles, for our participants. As they inevitably evaluated the racist attitudes, beliefs, and behaviours that they encountered as negative, it was impossible for them to assimilate white people’s perceptions into their existing identities or modify their existing identities to accommodate them in any positive way.

The first theme revealed how a pervasive sense of alienation and insecurity diminished participants’ feelings of *belonging* and *continuity.* Belonging is considered a basic human need [57], and lack of belonging is associated with problems in social and psychological functioning such as anxiety and depression [58]. Analysis of survey data from veterinarians in Canada [59] found that feelings of belonging to their team were directly related to wellbeing, while feelings of belonging to the profession indirectly contributed to wellbeing through enhancing meaningful work experiences. Analysis of interview data from UK veterinary students [60] found that a sense of social, academic, and professional belonging enhanced their wellbeing and career preparedness. Our participants reported a more profound lack of belonging in that they not only felt isolated from their teams and from the veterinary profession, but realised that they were perceived by white people as anomalous and unassimilable outsiders who could never be truly British. Indeed, a key narrative in the data was that white people often equated ‘Britishness’ with whiteness, thus excluding BAME people regardless of their place of birth, citizenship, identity, integration into, or contributions to, British society. This is a well-documented type of exclusionary discourse and racialised construction of identity and belonging, explored for example by Gilroy [61]. It is rooted in historical, colonial narratives [62], and continues to affect modern perceptions of who is truly ‘British’. Our participants experienced ‘social abjection’ [63] when their Britishness was questioned or denied based on their skin colour. This loss of national identity also caused some to lose their sense of continuity, that is, the persistence of an integrated and coherent identity over time. This is considered vital to psychological wellbeing; while high self-continuity is linked to life satisfaction and positive behavioural outcomes, low self-continuity is strongly associated with negative mood, suicide ideation, and symptoms of psychopathology [64].

The second theme examined how being perceived as homogenous and inferior undermined participants’ sense of *distinctiveness, self-esteem,* and *self-efficacy.* The tendency for white people to see them as different from members of their white ingroup (intergroup contrast), but also as indistinguishable members of a homogenous outgroup rather than unique individuals (intragroup similarity), is a robust and widespread phenomenon known as the ‘outgroup homogeneity effect’ [65]. Members of groups with lower social positions (e.g., small, subordinate, or low-status groups) are more likely to be seen as deindividualised and interchangeable by members of groups with higher social positions, and individuals regarded as belonging to homogenised groups are subject to more stereotyping and prejudice [65]. This is a significant problem for BAME people in general, and the stereotypes experienced by our participants were largely typical ones of BAME people being morally, physically, and mentally inferior [49, 50, 66]. These damaged participants’ feelings of competence and self-worth, increased their anxiety about making mistakes, and often led them to use strategies that took an arduous physical and emotional toll, as identified in other studies [49, 50, 66], such as working much harder than their white colleagues.

The third theme explored how participants’ sense of disappointment and futility led to a loss of *meaning* in their lives. Meaning is widely considered to be critical for human wellbeing and flourishing [67], and its absence is linked to anxiety, hopelessness, apathy, depression, and suicide [68]. A theoretical overview of ‘meaning in life’ [69] outlined three dimensions: coherence (a sense of comprehensibility and consistency); purpose (a sense of core goals, aims, and direction); and significance (a sense that life is inherently valuable and worth living). Racism experienced in the veterinary sector threatened all of these for our participants. Instead of experiencing coherence, they encountered uncertainty, inconsistency, and disruption: some were uncertain about whether they should enter or stay in the profession; others realised how inconsistent white people’s understandings of racism and its effects were with their own; and for many, job satisfaction and lives beyond work were disrupted by feelings of insecurity, frustration, and anxiety. Instead of experiencing purpose, many felt that their work had become unrewarding and unfulfilling: their desire to be respected members of the profession seemed unattainable; their motivation for work had diminished; and they were left with a sense of pointlessness and defeat. Instead of experiencing significance, many endured a sense of devaluation and worthlessness: they were not appreciated in the same way as their white colleagues or peers; they did not feel to be of much importance to the world; and they could not achieve ‘eudaimonia’, an ancient Greek term for living successfully and responsibly in an intrinsically satisfying way [69]. Cake et al. [70] argued that eudaimonia is central to veterinarians’ experiences of long-term wellbeing as it enables them to maintain passion for their work, develop resilience in the face of challenges, and mature into their best possible selves.

Overarching all of these themes was the theme of identity conflict, defined by IPT as a form of identity threat. This issue was briefly considered by Chung and Armitage-Chan [18], as some of their student participants experienced clashes between cultural beliefs and veterinary duties (e.g., working on cattle farms despite their Hindu beliefs that cattle are sacred), or between white students’ views and their own (e.g., student peers rejecting their food as strange and inedible). We found identity conflict to be more complex and multifold, however. On an interpersonal level, there was conflict between participants’ own sense of identity (e.g., British national, confident and competent person, unique individual) and white people’s perceptions of them (e.g., outsider, unreliable and inferior person, interchangeable member of a homogenous group). White people also tended to disrespect the ethnic or cultural parts of participants’ identities, for example by assigning them nicknames rather than making the effort to pronounce their names. Participants became aware of a chasm between their own worldviews and those of white people; recognition that people who engaged in racist discourse or actions did not always intend to be offensive actually widened this rift by exposing the extent of ignorance and unwillingness to develop awareness of racism in UK society.

On an individual level, racism created internal conflict in many participants. They began to doubt their abilities or question who they really were, as ongoing racism ‘changes your view of yourself’ (P17). Some felt obliged to try to change to ‘fit the mould’ (P65), despite knowing that this was ultimately neither possible nor desirable. There was tension between the identities they felt compelled to project to others and those they believed to be genuine. They sometimes felt alienated from their previous selves, and struggled to imagine who they would become in future. Many implied that their identities were being diminished, distorted, or destroyed, sometimes by gradual invalidation and destabilisation but sometimes by brutal destruction, expressed in statements such as, ‘it completely broke me’ (P04), ‘it feels like a ton of bricks has landed on you’ (P17), and ‘it shreds me from within’ (P36). The expression of being shredded evokes a particularly powerful image of the shattering psychological impact of racism, implying that the participant’s sense of identity had been reduced to tiny, useless fragments that could never be restored to a whole. Experiencing fragmentation of the self is a symptom of post-traumatic stress disorder [71], and McGee et al. [66] would describe some participants’ experiences as ‘racial battle fatigue’, a form of debilitating psychological and physiological stress caused by racial trauma [72].

This identity conflict may be exacerbated in professions regarded as ‘vocations’ or ‘callings’; people are often deeply committed to veterinary work [73], and many veterinary students have held their career ambitions from an early age [60, 74]. This mirrors other public-facing professions dedicated to social responsibility, such as medicine and teaching, as doctors and teachers frequently express fervour for their work, finding it to be inspiring and meaningful (e.g., [75, 76]). In such careers, development of a strong professional identity, in which personal and professional values are fully integrated and consistent, contributes substantially to a sense of self [77]; in medicine, for example, it has been argued that ‘authentic and unshakable incorporation’ of personal and professional identities occurs ([78], p. 243), and veterinary professionals have similarly recognised their professional identities as being central to how they define themselves [74]. Experiencing racism in such professions may have a more detrimental impact on people’s sense of identity than in professions involving less profound personal commitment.

To what extent can these findings be applied beyond the UK veterinary profession? Although the individual incidents reported by our participants were context-specific, the types of racism encountered and the wide spectrum of negative emotions they evoked are well-documented elsewhere (e.g., [44–46, 56]. This suggests that the UK veterinary sector does not generate atypical or unexpected forms of racism, but appears to provide an environment in which deep-rooted and pervasive forms of racism in contemporary Western societies remain more overlooked, ignored, and tolerated than in many other UK professions today. The novel aspect of our study, application of the theoretical framework of IPT to gain deeper insight into the psychological impact of racism, could therefore be relevant for understanding and addressing this problem and its consequences on a much wider scale.

Our findings have implications for the design and monitoring of real-world interventions. However, three main limitations should be taken into account. First, the data comprised relatively brief responses to survey questions. Although this meant that participants remained anonymous and unbiased by the social contexts of face-to-face interviews or focus groups, it is unlikely that the full nature of their experiences was captured. Further research could use interviews to gain deeper and more detailed understanding, by facilitating participant reflection on their experiences. Second, our analysis did not distinguish between ethnic groups within the broad BAME category. This enabled a diverse overview of encounters with racism, but may have masked disparities between BAME sub-groups, especially as Asian and Asian British participants predominated. The term BAME, along with other collective terms such as ‘non-white’ or ‘minority ethnic’, is frequently criticised for its limitations, especially because it appears to homogenise heterogenous social groups [79]. However, it has also been acknowledged that without a collective term, attempts to highlight and address institutional and structural racism would be diluted and fragmented [79]. As we sought to explore an under-researched topic and raise awareness of the magnitude and scope of racism experienced in the veterinary sector, we focused on the shared experiences of a broad sample of participants. Also, while we recognise that an intersectional approach to the analysis would have helped further situate participants’ experiences of racism within the context of their gender and professional identities, this would have risked compromising anonymity. As such, in this analysis we have not investigated potential differences in the nature of racism experienced by different BAME groups or genders. Future studies could focus on specific ethnic groups, and explore issues such as the dual discrimination reported by some female participants. Third, examination of the coping strategies used by participants, or the factors that they found to be supportive, fell beyond the scope of this study. Given the psychological distress participants experienced as a result of racism, future research on the ways in which BAME people and allies can challenge racism may be of value, but not without continued examination of the systemic changes required to eliminate the conditions that perpetuate it. A dual approach is required to acknowledge and highlight the broader societal responsibility to dismantle systemic racism, and to help empower those affected by it.

## Conclusions

Our findings highlight the urgent need to raise awareness of and tackle racism experienced in low ethnic diversity professions such as veterinary medicine and nursing, in the UK and beyond. By raising awareness of all forms of racism, initiating stringent anti-racism policies, and developing interventions to support and protect BAME people at all stages of their training and careers, such professions could reposition themselves as strong and commendable examples for others to follow.

## Supplementary Information

Supplementary Material 1.

## Data Availability

Survey materials are available from the corresponding author on request. However, due to the nature of the research and the potential for individuals from minority groups in a small profession to be identifiable, we are not able to share the raw data for ethical reasons.

## Abbreviations

BAME: Black, Asian, and minority ethnic
BVA: British Veterinary Association
BVEDS: British Veterinary Ethnicity and Diversity Society
IPT: Identity Process Theory
RCVS: Royal College of Veterinary Surgeons
UK: United Kingdom
URN: Unique reference number
US: United States

## Footnotes

1 The authors are aware of the limitations of the acronym BAME, but it is used in this study due to the absence of a clearer and more widely-accepted alternative in the UK.

2 The authors are aware that people from white minority ethnic groups can also experience discrimination, but our study focuses on People of Colour.
