# Supplementary material for: “It shreds me from within”: thematic analysis of the impact of racism on veterinary professionals and students in the United Kingdom
Source: BMC Psychol. 2025 May 31;13:585. doi: 10.1186/s40359-025-02481-x (PMC12125884; doi:10.1186/s40359-025-02481-x)
Supplement: Supplementary file 1 — Supplementary Material 1. [file 40359_2025_2481_MOESM1_ESM.docx]

**Experiences of racism and its impacts on mental wellbeing in Black, Asian and Minority Ethnic (BAME) people working and studying in the UK veterinary sector**

**Information for Participants**

**Welcome to our survey**

The purpose of this study is to gain understanding of the experiences and perceptions of racism in Black, Asian and Minority Ethnic people working or studying in the UK veterinary sector. In order to inform development and monitor the effectiveness of appropriate interventions to tackle racism and increase diversity in the sector, it is necessary to understand people’s experiences and perceptions of all types of racism.

**Who is eligible to take part in this study?** Black, Asian and Minority Ethnic people working or studying in any part of the UK veterinary sector are eligible to take part, including people in any clinical, non-clinical or support role in veterinary organisations, institutions, industry or practices.

**What does taking part involve?** Taking part in this study involves completion of a questionnaire, which includes writing an account of your experiences of racism encountered whilst working/studying in the UK veterinary sector.

- You will first be asked to answer eight demographic ‘tick box’ questions.
- You will then be asked to answer six further ‘tick box’ questions and two ‘free text’ questions to tell us about your experiences and perceptions of racism while studying or working in the veterinary sector, and the impact that this has had on you. There are also two optional ‘free text’ questions that allow you to make any suggestions about what you think could or should be done to help tackle racism in the veterinary sector, and provide any additional comments.

It would be useful to us if you can provide as much detail as you feel comfortable providing when writing about your experiences, as this helps with our qualitative analyses.

We are also planning to conduct a small number of follow-up telephone or video call interviews with selected participants to explore their experiences further.

- If you are willing to be contacted about a follow-up interview you will be able to provide contact information at the end of the questionnaire.
- This is not a commitment - you will not be under any obligation to proceed with an interview if you change your mind.

**Is participation in this study voluntary?** Yes. If taking part upsets you in any way, we encourage you to contact sources of support provided throughout the questionnaire.

**Is this study anonymous?**  Yes. Interview volunteers will be asked to provide a contact method such as an email address or phone number, but are not required to give their name. In accordance with the General Data Protection Regulation 2018, we will not use these contact details for any other purpose and will not share them with anyone else. Once the study is completed, contact details will be permanently deleted.

**How will the information I provide be used?** The accounts provided will be qualitatively analysed and findings will be presented at scientific conferences, to veterinary professional bodies, and published in an academic journal. Reports and presentations will include selected direct quotes from participants’ accounts. We will remove or change any potential identifiers of any individual or organisation from the quotes we use.

**Can I withdraw from this study at any time?** You are able to withdraw from this study at any time prior to submitting your questionnaire responses. However, once submitted, anonymised data cannot be withdrawn. Interview participants will be able to withdraw their data, without giving a reason, at any point until the final research report is submitted.

**Does this study have ethical approval?** Yes. Ethical approval has been granted by the Social Science Research Ethical Review Board at the Royal Veterinary College, URN SR2020-0224.

**If you have any further questions about this study, please contact:**

Victoria Crossley, Royal Veterinary College, [vcrossley@rvc.ac.uk](mailto:vcrossley@rvc.ac.uk) or

Navaratnam Partheeban, British Veterinary Ethnicity and Diversity Society, navaratnam.partheeban@pahc.com

**Sources of support:**

- Vetlife (Helpline: 0303 040 2551) <https://www.vetlife.org.uk/how-we-help/vetlife-helpline/>
- British Veterinary Ethnicity and Diversity Society <https://bveds.com/>
- Samaritans (08457 909090) [www.samaritans.org/](http://www.samaritans.org/)

**Prev / Next**

**Experiences of racism and its impacts on mental wellbeing in Black, Asian and Minority Ethnic (BAME) people working and studying in the UK veterinary sector**

**Statement of Consent to take part**

- I confirm that I have read and understood the Information for Participants for this study. I have had the opportunity to consider the information and ask questions which have been answered to my satisfaction.
- I understand that anonymised data cannot be withdrawn from the study, once submitted.
- I consent to the processing of my information for the purposes explained in the Information for Participants.
- I understand that details of my anonymised account may be presented at conferences and in research publications.

**1.* I agree to all of the above**

**□** Yes (must be ticked in order to proceed)

**□** No

**Prev / Next**

**Experiences of racism and its impacts on mental wellbeing in Black, Asian and Minority Ethnic (BAME) people working and studying in the UK veterinary sector**

**Questionnaire – Part 1 of 3**

**2.* What is your ethnicity?**

- **□** Indian
- **□** Pakistani
- **□** Bangladeshi
- **□** Chinese
- **□** Any other Asian or Asian British ethnicity (please specify below)
- **□** African
- **□** Caribbean
- **□** Any other Black or Black British ethnicity (please specify below)
- **□** White and Black Caribbean
- **□** White and Black African
- **□** White and Asian
- **□** Any other Mixed or Multiple ethnicity (please specify below)
- **□** Arab or British Arab
- **□** Any other ethnic group (please specify below)
- **□** Prefer to self-define (please specify below)

[free text 50 character comment box to enter any of the above labelled ‘please state where indicated by the options above’]

**3.* What gender do you identify as?**

- **□** Female
- **□** Male
- **□** Other gender identity (please specify below)
- **□** Prefer not to disclose

[free text comment box stating ‘please specify where have indicated ‘other gender identity’]

**4.* How old are you?**

- **□** <25
- **□** 26-35
- **□** 36-45
- **□** 46-55
- **□** 56-65
- **□** >66
- **□** Prefer not to disclose

**5.* What is your current predominant role in the veterinary sector?**

- **□** Qualified veterinary surgeon (clinical role)
- **□** Qualified veterinary surgeon (non-clinical role)
- **□** Registered veterinary nurse (clinical role)
- **□** Registered veterinary nurse (non-clinical role)
- **□** Veterinary student
- **□** Student veterinary nurse
- **□** Prefer not to disclose
- **□** Other role (please specify below)

[free text comment box stating ‘please specify where have indicated ‘other role’]

**6.* In which part of the veterinary sector do you currently work/study?**(If you work/study in more than one sector, please indicate the predominant one)

- **□** Private practice (independent)
- **□** Private practice (corporate)
- **□** Charity
- **□** University/Academia
- **□** Industry
- **□** Prefer not to disclose
- **□** Other (please specify)

**7.* If you work in practice, what type of practice do you work in?**

- **□** Companion/small animal
- **□** Production/large animal
- **□** Equine
- **□** Mixed
- **□** Prefer not to disclose
- **□** Not applicable, I do not work in practice
- **□** Other (please specify)

**8.* In which UK region do you currently predominantly work/study?**

- **□** East
- **□** East Midlands
- **□** London
- **□** North East
- **□** North West
- **□** Northern Ireland
- **□** Scotland
- **□** South East
- **□** South West
- **□** Wales
- **□** West Midlands
- **□** Yorkshire And The Humber
- **□** Prefer not to disclose

**9.* Is the geographical area in which you currently work/study predominantly rural, urban or suburban?**

- **□** Rural
- **□** Urban
- **□** Suburban
- **□** Prefer not to disclose
- **□** Other (please specify)

**Sources of support:**

- Vetlife (Helpline: 0303 040 2551) <https://www.vetlife.org.uk/how-we-help/vetlife-helpline/>
- British Veterinary Ethnicity and Diversity Society <https://bveds.com/>
- Samaritans (08457 909090) [www.samaritans.org/](http://www.samaritans.org/)

**Prev / Next**

**Experiences of racism and its impacts on mental wellbeing in Black, Asian and Minority Ethnic (BAME) people working and studying in the UK veterinary sector**

**Questionnaire – Part 2 of 3**

**10.* To what extent have you experienced ‘everyday racism’ within the context of the veterinary sector?** *‘Everyday racism’ refers to the systemic, commonplace interactions with people, services or systems that intentionally or unintentionally leaves individuals feeling racially judged in a covert or deniable way. This includes ‘racial microaggressions’ – which are the (sometimes subtle) forms of bias, damaging attitudes, behaviours, humiliations and jokes that people from minority groups face on a daily basis.*

- **□** Never
- **□** Rarely
- **□** Sometimes
- **□** Frequently
- **□** Very frequently

**11.* To what extent have you experienced more extreme incidents of racism within the context of the veterinary sector?** *More extreme incidents of racism includes incidents of frank discrimination, overt racism and/or racist attacks.*

- **□** Never
- **□** Rarely
- **□** Sometimes
- **□** Frequently
- **□** Very frequently

**12.* How frequently have you experienced incidents of racism from clients within the context of the veterinary sector?** *This includes any form of racism, including everyday racism or more extreme incidents.*

- **□** Never
- **□** Rarely
- **□** Sometimes
- **□** Frequently
- **□** Very frequently
- **□** Not applicable – my work/study in the veterinary sector does not involve contact with clients

**13.* How frequently have you experienced incidents of racism from other people who are also working or studying in the veterinary sector, such as colleagues and/or peers?** *This includes any form of racism, including everyday racism or more extreme incidents.*

- **□** Never
- **□** Rarely
- **□** Sometimes
- **□** Frequently
- **□** Very frequently

**14.* Have your experiences of racism whilst working/studying in the veterinary sector affected your wellbeing or mental health?** *This includes any form of racism, including everyday racism or more extreme incidents.*

- **□** No, not at all
- **□** Yes, slightly
- **□** Yes, moderately
- **□** Yes, considerably
- **□** Yes, extremely
- **□** Not applicable

**15.* Have your experiences of racism whilst working/studying in the veterinary sector affected your satisfaction with your job and/or studies?** *This includes any form of racism, including everyday racism or more extreme incidents.*

- **□** No, not at all
- **□** Yes, slightly
- **□** Yes, moderately
- **□** Yes, considerably
- **□** Yes, extremely
- **□** Not applicable

**Sources of support:**

- Vetlife (Helpline: 0303 040 2551) <https://www.vetlife.org.uk/how-we-help/vetlife-helpline/>
- British Veterinary Ethnicity and Diversity Society <https://bveds.com/>
- Samaritans (08457 909090) [www.samaritans.org/](http://www.samaritans.org/)

**Prev / Next**

**Experiences of racism and its impacts on mental wellbeing in Black, Asian and Minority Ethnic (BAME) people working and studying in the UK veterinary sector**

**Questionnaire – Part 3 of 3**

**Please take a few minutes to reflect on your own experiences of any type of racism you have encountered while working and/or studying in the veterinary sector in the UK. We would be grateful if you could then write an account of your experiences, guided by the four text boxes provided below.**

Rather than providing some established definition of racism, we are interested to hear about anything you regard as racism, including your experiences of ‘everyday racism’, microaggressions, or other common behaviours, however minor, as well as any more extreme incidents.

- The first two text boxes ask for information about: i) details of the incident(s), and ii) the impact the experience(s) had on you.

In the last two text boxes, we are also interested in any suggestions you would like to make about iii) what you think could or should be done to tackle racism in the veterinary sector, and iv) any other information you think is relevant.

**16.* Please describe in detail any racist incidents you have witnessed or experienced in the context of the veterinary sector.** *Relevant details might include, for example, the types or roles of the people involved, when (how long ago) and where the incident(s) occurred, whether you reported the incident(s) or sought support, and the outcome of any report(s) made. (Please write as much as you are comfortable to - including detail helps with our qualitative analysis. There is no word limit.)*

[free text box….]

**17.* Please describe how you felt, how you dealt with these incident(s), and any impact this has had on your wellbeing, mental health, or job satisfaction.** *(Please write as much as you are comfortable to - including detail helps with our qualitative analysis. There is no word limit.)*

[free text box….]

**18. If you have any suggestions about what you think could or should be done to help tackle racism in the veterinary sector, please enter these in the text box below.**

[free text box….]

**19. Please use the text box below to add any additional comments you feel may be relevant.**

[free text box….]

**Sources of support:**

- Vetlife (Helpline: 0303 040 2551) <https://www.vetlife.org.uk/how-we-help/vetlife-helpline/>
- British Veterinary Ethnicity and Diversity Society <https://bveds.com/>
- Samaritans (08457 909090) [www.samaritans.org/](http://www.samaritans.org/)

**Prev / Next**

**Experiences of racism and its impacts on mental wellbeing in Black, Asian and Minority Ethnic (BAME) people working and studying in the UK veterinary sector**

**Volunteering for a follow-up interview**

**We are planning to contact a small number of participants for a follow-up interview, to explore their experiences of racism further.**

This would be in the form of a telephone conversation or video call at the participant’s convenience and would take up to one hour. Should you opt to be contacted, you will also be free to decline to be interviewed if you have since changed your mind.

20.**If you are willing to be contacted about a possible follow-up interview, please indicate your preferred contact method by providing your email address and/or telephone or mobile number. If you do not wish to be contacted, please leave these boxes blank.**

- - Email [text box]
  - Telephone call [text box]
  - Text message [text box]
  - If you have any preferences with regard to time of day to be contacted, please enter these here [free text box]

**Sources of support:**

- Vetlife (Helpline: 0303 040 2551) <https://www.vetlife.org.uk/how-we-help/vetlife-helpline/>
- British Veterinary Ethnicity and Diversity Society <https://bveds.com/>
- Samaritans (08457 909090) [www.samaritans.org/](http://www.samaritans.org/)

**Prev / Next**

**Experiences of racism and its impacts on mental wellbeing in Black, Asian and Minority Ethnic (BAME) people working and studying in the UK veterinary sector**

**Thank you very much for your participation.**

**To submit your answers, please click 'Done' at the bottom of this page**

The purpose of this study was to gain understanding of the experiences and perceptions of racism in Black, Asian and Minority Ethnic people working or studying in the veterinary sector in the UK. It is necessary to understand people’s experiences and perceptions of all types of racism, in order to inform development of appropriate interventions to tackle racism and monitor their effectiveness.

This is an anonymous study which has been ethically approved. The findings will be presented at scientific conferences, to veterinary professional bodies, and published in an academic journal. Reports and presentations will include selected direct quotes from participants’ accounts, but we will remove or change any potential identifiers of any individual or organisation from the quotes we use.

If you have any further questions about this study, you are welcome to email us:

Victoria Crossley (Royal Veterinary College) vcrossley@rvc.ac.uk

Navaratnam Partheeban (British Veterinary Ethnicity and Diversity Society) navaratnam.partheeban@pahc.com

**If engaging in this study has upset or distressed you in any way, please do not hesitate to contact the sources of support below.**

**Sources of support:**

- Vetlife (Helpline: 0303 040 2551) <https://www.vetlife.org.uk/how-we-help/vetlife-helpline/>
- British Veterinary Ethnicity and Diversity Society <https://bveds.com/>
- Samaritans (08457 909090) [www.samaritans.org/](http://www.samaritans.org/)

**Prev / Done**
